# Comparison of Two Methods for Weaning from Nasal Continuous Positive Airway Pressure via the Cyclic Use of High-Flow Nasal Cannula or Room Air in Preterm Infants

**DOI:** 10.3390/children11030351

**Published:** 2024-03-15

**Authors:** Shu-Ting Yang, Hao-Wei Chung, Hsiu-Lin Chen

**Affiliations:** 1 Division of Neonatology, Department of Pediatrics, Kaohsiung Medical University Hospital, Kaohsiung Medical University, Kaohsiung 807, Taiwan; 1000410@kmuh.org.tw (S.-T.Y.); 1000461@kmuh.org.tw (H.-W.C.); 2 Department of Respiratory Therapy, College of Medicine, Kaohsiung Medical University, Kaohsiung 807, Taiwan

**Keywords:** nasal continuous positive airway pressure, high-flow nasal cannula, preterm

## Abstract

Nasal continuous positive airway pressure (NCPAP) is extensively used for preterm infants experiencing respiratory distress syndrome (RDS). Weaning from NCPAP includes direct weaning or gradually extending room air exposure. However, a high-flow nasal cannula (HFNC) is an alternative weaning method. Therefore, this study evaluated the clinical outcomes of HFNC and progressively increasing room air duration as weaning strategies. This study enrolled 46 preterm infants with RDS receiving NCPAP support who underwent the cyclic use of NCPAP and HFNC weaning protocol as the HFNC group; a retrospective analysis included 87 preterm infants weaned from NCPAP by gradually extending room air duration as the room air group. Differences in clinical conditions, complications, and short-term outcomes between the weaning methods were compared. The mean post-menstrual age at initiating NCPAP weaning was lower in the room air group than in the HFNC group (mean ± SD, 35.2 ± 2.3 weeks vs. 33.2 ± 2.5 weeks, *p* < 0.001). Hospital stay duration and total respiratory therapy days were longer in the HFNC group (96 ± 38 days and 80 ± 37 days, respectively) than in the room air group (78 ± 28 days and 56 ± 25 days, respectively), with *p*-values of 0.006 and <0.001. In conclusion, employing HFNC for weaning from NCPAP resulted in longer hospital admissions and respiratory therapy days than the room air method. However, further studies with a larger sample size are warranted for a more comprehensive evaluation, given the limited number of enrolled patients.

## 1. Introduction

Respiratory distress syndrome (RDS) is a common cause of admission for preterm infants to neonatal intensive care units (NICUs). Non-invasive respiratory support for preterm infants includes the use of nasal intermittent positive pressure ventilation (NIPPV), nasal continuous positive airway pressure (NCPAP), and high-flow nasal cannula (HFNC). NCPAP has been extensively used as a non-invasive respiratory therapy for preterm infants experiencing RDS. Currently, the main clinical methods for weaning from NCPAP include direct weaning, gradually tapering the setting of positive end-expiratory pressure (PEEP), and progressively extending room air exposure duration. However, a definite conclusion regarding the superior method for weaning preterm infants from NCPAP is still lacking [1]. HFNC provides PEEP using high-flow oxygen (2–8 L/min) for infants [2,3,4], and it has been considered an alternative method for weaning preterm infants from NCPAP [2,3,4]. However, its efficiency compared to weaning from NCPAP by gradually tapering down the NCPAP setting remains uncertain. In our NICU, we have employed a cyclic approach using NCPAP and room air to gradually wean preterm infants from NCPAP support for years. If infants demonstrate tolerance to room air, they remain on it throughout the day. We have also used HFNC for NCPAP weaning purposes since 2019. Therefore, this study aimed to evaluate the clinical outcomes of both methods for weaning from NCPAP, involving HFNC and progressively increasing room air duration.

## 2. Materials and Methods

### 2.1. Study Population

The prospective observational study enrolled 46 preterm infants (gestational age [GA] < 37 weeks) with RDS using NCPAP (Babi. Plus^®^ Bubble CPAP, Gale Med corporation, Taipei, Taiwan) support who underwent weaning from NCPAP between June 2019 and April 2021 in the NICU at Kaohsiung Medical University Hospital (KMUH) as the HFNC group. In contrast, a retrospective study enrolled 87 preterm infants weaned from NCPAP by progressively extending room air duration between January 2016 and December 2018 in the NICU at KMUH as the room air group. The term “room air” referred to the condition where infants were free from any form of non-invasive ventilatory support. The evaluation criteria for weaning from NCPAP were consistent across both groups, including a fraction of inspired oxygen (FiO_2_) of 0.21, PEEP of 4–5 cm H_2_O, relatively stable vital signs, and absence of apnea with NCPAP support for 3 days. Additionally, subjective clinical judgment for weaning NCPAP was made through intensive observation of preterm infants’ condition, involving NICU nurses and respiratory therapists in both groups. Successful weaning from NCPAP to room air or from HFNC to room air was defined as stability in room air without requiring any respiratory support for 7 consecutive days. The target oxygen saturation was set at 91–95% [5].

We recorded primary and antenatal data, including GA, birth body weight (BBW), sex, type of birth (vaginal delivery or cesarean section), Apgar scores at 1 and 5 min after birth, the Neonatal Therapeutic Intervention Scoring System (NTISS) [6], RDS grade, intubation after birth (the indication of intubation after birth was according to the Neonatal Resuscitation Program^®^) [7], medications for apnea (theophylline or caffeine), packed red blood cell transfusion, moderate and severe bronchopulmonary dysplasia, patent ductus arteriosus (PDA) with conservative treatment or without treatment, late-onset sepsis (blood culture-proven or clinically diagnosed), hypotension (defined as a mean arterial blood pressure below the gestational age or the post-menstrual age of the infant), intraventricular hemorrhage ≥ grade III, retinopathy of prematurity (ROP) ≥ stage III, and necrotizing enterocolitis (NEC) ≥ stage II. Respiratory outcomes included: length of hospital stay at initiating NCPAP weaning; post-menstrual age (PMA) at the initiation of NCPAP weaning; duration of weaning from NCPAP to HFNC or room air in the HFNC and room air groups, respectively; PMA at the discontinuation of NCPAP; duration from the initiation of NCPAP weaning to achieving complete room air weaning; length of hospital stay; PMA at discharge; and total respiratory therapy days.

### 2.2. Methods of NCPAP Weaning

#### 2.2.1. HFNC Group

These 46 preterm infants in the HFNC group underwent the cyclic use of NCPAP and HFNC weaning protocol (Fisher & Paykel Opti flow System, Healthcare, Auckland, New Zealand) every 3 h (i.e., using NCPAP for 3 h, followed by switching to HFNC for 3 h, and subsequently continuing to alternate consecutively) for 3 days in the NICU at KMUH. The evaluation criteria for weaning from NCPAP to HFNC included maintaining a fraction of FiO_2_ of 0.21, PEEP of 4–5 cm H_2_O, relatively stable vital signs, and the absence of apnea with NCPAP support for 3 days. The flow rate of HFNC was adjusted to match the pressure level during HFNC, as measured using a GiO Digital Pressure Gauge (GIO 6, GaleMed Corporation, Taipei, Taiwan) from the distal end of the nasal cannula. The pressure was set at the same level as the PEEP of the preceding NCPAP. The flow rate of HFNC was maintained at 4–6 L/minute throughout the study. During the cyclic use of NCPAP and HFNC, we changed the CPAP driver and the nasal attachments for HFNC precisely every 3 h during the weaning process. This cyclic approach was selected based on the standard nursing care schedule in our NICU, which operates on a 3 h cycle. Additionally, we anticipated that providing respiratory support under HFNC for 3 h intervals would offer more stable support to preterm infants compared to shorter duration cycles. Once patients achieved stability, we allowed them to transition directly or cyclically from HFNC to room air based on their individual condition.

#### 2.2.2. Room Air Group

In the room air group, the weaning method in the NICU at KMUH involved the cyclic use of NCPAP and room air in 3 h intervals, gradually increasing the duration of time spent off NCPAP in 3 h intervals. For instance, once the preterm infant achieved clinical stability, we attempted to remove NCPAP for 1 h within the 3 h interval, with the remaining 2 h using NCPAP. If the preterm infant tolerated this period without NCPAP (indicated by the absence of tachypnea as a respiratory rate exceeding 60 cycles per minute, oxygen desaturation, and no increase in apnea frequency), we extended the duration of time spent in room air the following day (for example, 2 h without NCPAP and 1 h using NCPAP within the 3 h interval) [8].

### 2.3. Study Design

This study evaluated and compared the differences in clinical conditions, complications, and short-term outcomes of preterm infants between the two weaning strategies.

### 2.4. Statistics Analyses

Data recording and evaluation were performed using JMP 10 software (SAS Institute Inc., Cary, NC, USA). A paired *t*-test was used to compare the numerical variables of the characteristics and clinical outcomes of the preterm infants between the HFNC and room air groups. Univariate and multiple regression analyses were used to analyze the factors influencing the outcomes of preterm infants in the HFNC and room air groups.

### 2.5. Ethics Approval

These prospective observational and retrospective study protocols were approved by the Human Experiment and Ethics Committee of KMUH (approval numbers: KMUHIRB-SV(I)-20180059 and KMUHIRB-SV(II)-20210106, respectively). Written informed consent was obtained from the parents of all included preterm infants. All experiments were performed in accordance with the relevant guidelines and regulations.

## 3. Results

In the HFNC group, 46 preterm infants (27 males and 19 females) were admitted to the NICU at KMUH between June 2019 and April 2021. No adverse effects were observed during the study. In the room air group, 87 preterm infants (42 males and 45 females) admitted to the NICU at KMUH between January 2016 and December 2018 were enrolled in the retrospective study. Table 1 presents the characteristics and clinical outcomes of the preterm infants in the HFNC and room air groups. No significant differences were found in GA (mean ± standard deviation: HFNC versus room air group, 28.7 ± 2.6 versus 28.4 ± 1.9 weeks, *p* = 0.433) and BBW (HFNC versus room air group, 1181 ± 354 versus 1099 ± 240 g, *p* = 0.165) between the two groups. The overall administration rate of methylxanthines (including theophylline and caffeine) was 79% in the HFNC group and 92% in the room air group, with a *p*-value of 0.025. The decision to administer methylxanthines (either aminophylline or caffeine) for the treatment of apnea was determined based on the clinical condition of the infants. Therefore, we infer that the difference observed between the two groups was likely influenced by the clinical condition of the infants involved. The administration of methylxanthines, including theophylline or caffeine, for apnea of prematurity treatment was obviously different (HFNC versus room air group under caffeine treatment: 35% versus 15%, *p* = 0.001; under theophylline treatment: 43% versus 77%, *p* = 0.001). Since caffeine was introduced at KMUH in 2016, this may have caused a significant difference between the HFNC and room air groups in the proportion of preterm infants treated with caffeine therapy for apnea of prematurity. The other was the ratio of hypotension, which was higher in the HFNC group than in the room air group (26% versus 10%, *p* = 0.018).

Table 2 presents the respiratory outcomes and prognoses of the two weaning groups. The length of hospital stay at the initiation of NCPAP weaning from NCPAP was significantly lower in the room air group than in the HFNC group (48 ± 28 versus 34 ± 26 days, *p* = 0.004). The PMA at the initiation of weaning from NCPAP was significantly lower in the room air group than in the HFNC group (33.2 ± 2.5 versus 35.6 ± 2.3 weeks, *p* < 0.001) (Table 2). Additionally, the duration of weaning from NCPAP to HFNC in the HFNC group or to room air in the room air group was shorter in the HFNC group than in the room air group (7 ± 10 versus 20 ± 11 days, *p* < 0.001) (Table 2). No significant difference was found in PMA at the discontinuation of NCPAP between the two groups. The length of hospital stay (96 ± 38 versus 78 ± 28 days, *p* = 0.006) and the total respiratory therapy days (80 ± 37 versus 56 ± 25 days, *p* < 0.001) were significantly longer in the HFNC group than in the room air group.

Table 3 and Table 4 present univariate and multiple regression analyses of the factors that influenced PMA at the initiation of NCPAP weaning and the duration of weaning from NCPAP to HFNC in the HFNC group or to room air in the room air group, respectively. The factors affecting the PMA at the initiation of NCPAP weaning according to univariate regression analysis included the method of weaning from NCPAP, usage of surfactant, using theophylline treatment for apnea of prematurity, packed red blood cell transfusion, PDA with conservative treatment, and ROP ≥ stage III (Table 3). Multiple regression analysis revealed that the room air group was associated with shorter PMA at the initiation of NCPAP weaning (regression coefficient = −1.592, *p* = 0.002), and preterm infants with PDA undergoing conservative treatment was associated with longer PMA at the initiation of NCPAP weaning (regression coefficient = 0.961, *p* = 0.049) (Table 4). Univariate regression analysis revealed that the method of weaning from NCPAP, using theophylline treatment for apnea of prematurity, PDA with conservative treatment, and NEC ≥ stage II were factors influencing the duration of weaning from NCPAP to HFNC in the HFNC group or to room air in the room air group (Table 3). Furthermore, multiple regression analysis showed that the room air group (regression coefficient = 12.215, *p* < 0.001) and NEC ≥ stage II (regression coefficient = 21.569, *p* = 0.005) were associated with longer duration of weaning from NCPAP to HFNC in the HFNC group or to room air in the room air group (Table 4).

Table 5 and Table 6 show univariate and multiple regression analyses of the factors that influenced the duration from the initiation of NCPAP weaning to achieving complete room air and the total respiratory therapy days. Univariate regression analysis revealed that the NCPAP weaning method and intubation after birth might influence the duration from the initiation of NCPAP weaning to achieving complete room air (Table 5). Multiple regression analysis showed that the room air group (regression coefficient = −9.549, *p* = 0.001) and intubation after birth (regression coefficient = 5.778, *p* = 0.046) were associated with a shorter and longer duration from the initiation of NCPAP weaning to achieving complete room air, respectively (Table 6). Univariate regression analysis showed that the method of weaning from NCPAP, GA, BBW, sex, 1- and 5 min Apgar scores, RDS grade, surfactant usage, intubation after birth, medication for apnea of prematurity treatment, and ROP ≥ stage III were factors influencing the total respiratory therapy days (Table 5). Furthermore, multiple regression analysis revealed that the room air group (regression coefficient = −28.667, *p* < 0.001) had shorter total respiratory therapy days than the HFNC group and that higher GA (regression coefficient = −7.241, *p* < 0.001) and BBW (regression coefficient = −0.031, *p* = 0.004) were associated with shorter total respiratory therapy days (Table 6).

Table 7 and Table 8 present t univariate and multiple regression analyses of the factors that influenced the length of hospital stay and PMA at discharge. Univariate regression analysis revealed that the method of weaning from NCPAP, GA, BBW, sex, NTISS score, 1- and 5 min Apgar scores, RDS grade, intubation after birth, and ROP ≥ stage III were factors that might influence the length of hospital stay (Table 7). In contrast, multiple regression analysis revealed that the room air group (regression coefficient = −19.761, *p* < 0.001) and larger GA (regression coefficient = −4.424, *p* = 0.013) were associated with a shorter hospital stay, while higher NTISS scores (regression coefficient = 0.759, *p* = 0.023), intubation after birth (regression coefficient = 28.435, *p* < 0.001), and ROP ≥ stage III (regression coefficient = 12.059, *p* = 0.047) were associated with a longer hospital stay (Table 8). Additionally, univariate regression analysis revealed that the method of weaning from NCPAP, GA, BBW, sex, 1- and 5 min Apgar scores, RDS grade, medication for apnea of prematurity treatment, and ROP ≥ stage III were factors that might influence the PMA at discharge (Table 7). Multiple regression analysis revealed that the room air group (regression coefficient = −3.284, *p* < 0.001) and larger BBW (regression coefficient = −0.006, *p* < 0.001) were associated with shorter PMA at discharge (Table 8).

## 4. Discussion

Our study demonstrated the clinical conditions associated with the two NCPAP weaning methods. The mean PMA at the initiation of weaning was significantly lower in the room air group than in the HFNC group, while the mean PMA at weaning cessation showed no significant difference between the groups. Hospital stay duration and total respiratory therapy days were significantly longer in the HFNC group compared to the room air group. Additionally, the mean PMA at discharge was significantly lower in the room air group than in the HFNC group.

Various methods have been used for weaning preterm infants from NCPAP [1]. In our NICU, the weaning process for preterm infants involved gradually increasing room air exposure duration, beginning with 0.5 h under room air and 2.5 h under NCPAP in a cycle lasting for 3 h. Subsequently, the duration of room air exposure was gradually extended if the clinical condition of the preterm infants remained stable. However, this method was not compared with others, due to its limited application and publication in most NICUs for NCPAP weaning. Therefore, we compared the clinical outcomes between the room air and HFNC groups.

We regarded the use of HFNC every three hours as a progressive step in care, despite its potential lack of full individualization and shorter rest periods. Introducing an intermediate step like this can indeed be beneficial, even if it slightly prolongs the process. Such considerations are crucial in discussions of this nature. Therefore, we have adopted a cyclic approach, alternating between NCPAP with room air and HFNC, with a focus on ensuring the comfort of the infants. This decision was made recognizing that nasal prongs for NCPAP can cause discomfort by compressing the nostrils in our approach.

The PMA at the initiation of weaning from NCPAP was larger in the HFNC group than in the room air group, while the duration of weaning from NCPAP to HFNC in the HFNC group or to room air in the room air group was shorter in the HFNC group than in the room air group. Therefore, the PMA at the discontinuation of NCPAP was similar between the HFNC and room air groups (Table 2). This finding suggests that the PMA at the discontinuation of NCPAP may be similar, regardless of the weaning method employed. As for the weaning criteria, in both the HFNC and room air groups, the decision to initiate weaning from NCPAP was based on objective evaluation criteria. These criteria included maintaining a fraction of FiO_2_ of 0.21, PEEP of 4–5 cm H_2_O, stable vital signs, and absence of apnea with NCPAP support for 3 days. Additionally, decisions were influenced by the observations of NICU doctors, who noted a more stable clinical condition as reported by NICU nurses and respiratory therapists during routine care activities. These activities included respiratory care, sputum suctioning, diaper changing, and enteral feeding, allowing for the temporary cessation of NCPAP for preterm infants.

The higher ratios of surfactant usage and intubation after birth in the HFNC group compared to the room air group (50% and 43% vs. 38% and 28%, respectively) suggest potentially more severe respiratory conditions in the former, although statistical significance was not reached (*p* = 0.18 and 0.064, respectively). We hypothesize that these factors are indicative of more severe respiratory conditions in the HFNC group and may explain the larger PMA at the initiation of weaning from NCPAP observed in the HFNC group in our NICU. However, it is worth noting that HFNC may provide adequate pressure support to the respiratory tract compared to room air, which could contribute to the shorter duration of weaning from NCPAP to HFNC in the HFNC group compared to weaning to room air in the room air group. Therefore, the lack of significant differences in PMA between the two groups at the discontinuation of NCPAP could be attributed to these factors.

A few studies have evaluated the use of HFNC as an NCPAP weaning method. Badiee et al. compared 88 preterm infants who were weaned from NCPAP via HFNC or room air and found no significant differences in PMA at the discontinuation of NCPAP between the two groups, although the duration of weaning from NCPAP was shorter in the HFNC group than in the room air group [9]. The weaning protocol of Badiee et al. involved directly changing NCPAP to HFNC or room air, which differs from that used in our study [9]. Amatya et al. evaluated 68 preterm infants with GA between 26 and 32 weeks using gradually tapered-down PEEP and room air to wean them from NCPAP, and found no significant differences in PMA at the weaning cessation of NCPAP or the duration of weaning from NCPAP between the two methods; however, the success rate was higher in the NCPAP weaning group using gradually tapered down PEEP than in the group using room air [10]. The abovementioned studies used different methods to wean NCPAP compared to those employed in our study; therefore, the appropriate protocol for using HFNC to wean preterm infants from NCPAP remains uncertain.

We also observed that the duration from the initiation of NCPAP weaning to achieving complete room air, total respiratory therapy days, length of hospital stay, and PMA at discharge were shorter in the room air group than in the HFNC group (Table 2). Tang et al. conducted a study involving 60 preterm infants with a GA of <30 weeks to wean them from NCPAP and found that the shortest duration of weaning from NCPAP was achieved by a direct transition from NCPAP to HFNC support [11]. Abdel-Hady et al. conducted a survey of 60 preterm infants with a GA of >28 weeks and reported that the total respiratory therapy days were shorter when weaning from NCPAP was accomplished by gradually tapering down the setting of FiO_2_ than when it was achieved using HFNC. However, the success rate of weaning from NCPAP was not significantly different between the groups [12]. In a study involving 88 preterm infants, Badiee et al. discovered that the subgroups weaned from NCPAP via HFNC had a shorter hospital stay and total respiratory therapy days; however, they found no significant difference in the success rate of weaning from NCPAP between the groups [9]. Tang et al. surveyed 60 preterm infants with a GA of <30 weeks and found that the total respiratory therapy number was longer in the group weaned from NCPAP via HFNC. This prolonged duration was corrected by extending the time required by preterm infants to achieve complete oral feeding [11]. However, the abovementioned studies did not provide a definite conclusion regarding whether weaning from NCPAP via HFNC might prolong the number of respiratory therapy days or length of hospital stay. Habas et al. suggested that a prolonged use of HFNC could decrease the respiratory effort of preterm infants [13]. We hypothesized that besides the NCPAP weaning methods, other factors may influence PMA at the initiation of weaning from NCPAP, duration of weaning from NCPAP, duration from initiating weaning from NCPAP to achieving complete room air, total respiratory therapy days, length of hospital stay, and PMA at discharge. Therefore, we evaluated the factors associated with these outcomes using univariate and multiple regression analyses (Table 3, Table 4, Table 5, Table 6, Table 7 and Table 8).

We observed a significant relationship between preterm infants with PDA undergoing conservative treatment and PMA at the initiation of weaning from NCPAP (Table 4). Similarly, Rastogi et al. found that PDA may affect the time to successful NCPAP weaning in 454 preterm infants with a GA of <32 weeks [13]. A PDA requiring treatment induces significant hemodynamic changes in multiple organs, including the brain, lungs, and gastrointestinal tract, potentially prolonging the total respiratory therapy days in neonates [14]. Therefore, we concluded that preterm infants with PDA may exhibit more unstable respiratory conditions, thereby influencing PMA at the initiation of weaning from NCPAP, although no significant association was found in our study between PDA treated conservatively and the duration of weaning from NCPAP to HFNC in the HFNC group or to room air in the room air group (Table 4).

Our study also found that preterm infants diagnosed with NEC ≥ stage II had a longer duration of weaning from NCPAP to HFNC in the HFNC group or to room air in the room air group (Table 4). Similarly, Rastogi et al. reported that NEC may affect the duration of weaning from NCPAP [15]. NEC stages II and III, also known as definite NEC, represent life-threatening gastrointestinal conditions in preterm infants and are usually characterized by severe abdominal inflammation and critical changes in vital signs [16]. Consequently, we suspected that NEC ≥ stage II might influence the duration of weaning from NCPAP to HFNC in the HFNC group or to room air in the room air group because of the instability of vital signs associated with this condition. However, it is worth noting that we encountered only a few NEC cases in our NICU. According to data provided by the Taiwan Society of Neonatology from the Taiwan Neonatal Network between 2017 and 2019, the average incidence of NEC in our NICU was 1.5%, which is lower than the national average of 5% in Taiwan [17].

In our study, multiple regression analysis revealed a significant relationship between intubation after birth and the duration from the initiation of weaning from NCPAP to achieving complete room air (Table 6). Consistently, Rastogi et al. evaluated 454 preterm infants with a GA of ≤32 weeks receiving NCPAP support and found that those who required intubation after birth had a longer duration from the initiation of weaning from NCPAP to achieving complete room air [15]. Wilson et al. conducted a study involving 77,576 neonates, including both preterm and term infants, and discovered that neonates with poor birth conditions tended to have longer total respiratory therapy days [18]. Based on these findings, we inferred that preterm infants who required intubation after birth were likely to have more critical respiratory conditions at birth, necessitating more intensive respiratory therapy. This association may explain why these infants experienced a longer duration from the initiation of weaning from NCPAP to achieving complete room air because of their poor lung condition. Our study also revealed that a larger GA and BBW were significantly associated with shorter total respiratory therapy days (Table 6). Similarly, Wilson et al. found that a larger GA correlated with shorter total respiratory therapy days [18]. Typically, larger GA and BBW in preterm infants signify relatively stable clinical conditions and more advanced lung maturation, which may contribute to a shorter respiratory therapy duration.

We found that a lower GA, higher NTISS score, intubation after birth, and ROP ≥ stage III was significantly associated with a longer hospital stay (Table 8). This observation aligns with the findings of Murki et al., who surveyed 3095 preterm infants with a GA between 25 and 33 weeks and revealed that GA and BBW influenced the length of hospital stay [19]. Preterm infants with a lower GA usually experience poor clinical conditions after birth and are more prone to have more prematurity-related complications, which may contribute to a longer hospital stay. The NTISS score evaluates various aspects of a newborn’s condition after birth, including the respiratory system, monitoring needs, cardiovascular system, vascular access, metabolic and nutritional status, transfusion needs, major procedures, and drug therapies. A higher NTISS score indicates a more critical condition of the neonate after birth; therefore, it may correlate with a longer hospital stay. Intubation after birth suggests a more critical respiratory condition and may be associated with a longer hospital stay because of the longer duration from the initiation of weaning from NCPAP to achieving complete room air (Table 6). ROP is characterized by the abnormal growth of retinal vascular vessels and is predominantly observed in preterm infants who receive respiratory therapy with higherFiO_2_. Severe ROP cases may lead to retinal detachment, necessitating treatments such as retinal photocoagulation or the intravitreal injection of vascular endothelial growth factor inhibitors to prevent vision impairment [20]. Fortes et al. discovered that preterm infants with a GA of <32 weeks who required ventilator support were more susceptible to experiencing ROP [21]. Therefore, we hypothesized that preterm infants with severe ROP may have more unstable respiratory conditions and a higher fraction of inspired oxygen, which could influence the length of hospital stay. Lower BBW was found to be associated with longer PMA at discharge (Table 8). Similarly, Behera et al. reported that GA and BBW influenced PMA at discharge in preterm infants with a BBW of <1500 g [22]. Therefore, we inferred that preterm infants with a lower BBW had a longer PMA at discharge, possibly because of the longer total respiratory therapy days (Table 6).

Our study had some limitations. First, the sample size was small, which may have affected the analysis of the results and the generalizability of our findings. Second, the fact that HFNC devices were previously self-paid in Taiwan may have influenced their availability and use in our study. For this prospective observational study, the HFNC devices were funded by Kaohsiung Medical University Hospital (grant numbers: KMUH107-7M26 and KMUH107-7R44). Despite the introduction of HFNC to our NICU since 2015, the number of cases remained low due to the self-payment requirement. Nevertheless, we had prior clinical experiences with HFNC usage in the preterm and term infants before conducting this study. However, a larger sample size for further analyses should be considered in future studies, considering that the health insurance coverage for HFNC commenced in 2022 in Taiwan.

## 5. Conclusions

Our study found that weaning preterm infants from NCPAP using HFNC resulted in longer hospital stays and respiratory therapy days than using the cyclic room air method. The mean PMA at the initiation of weaning was significantly larger in the HFNC group, while the mean PMA at the discontinuation of NCPAP was similar between the HFNC and room air groups. We also observed that the duration from the initiation of weaning from NCPAP to achieving complete room air and PMA at discharge were lower in the room air group than in the HFNC group. However, further studies with a larger sample size are warranted for a more comprehensive evaluation of the effectiveness of different methods of weaning from NCPAP, given the limited number of enrolled patients in our study.

## Figures and Tables

**Table 1:** Characteristics and clinical outcomes of infants undergoing NCPAP weaning with HFNC and room air groups.

Item	HFNC Group (*n* = 46)	Room Air Group (*n* = 87)	*p* Value
GA (weeks) (mean ± SD)	28.7 ± 2.6	28.4 ± 1.9	0.433
BBW (g) (mean ± SD)	1181 ± 354	1099 ± 240	0.165
Male, *n* (%)	27 (59)	42 (48)	0.253
Vaginal delivery, *n* (%)	29 (63)	49 (56)	0.454
NTISS (mean ± SD)	19 ± 4	20 ± 8	0.737
1′ Apgar score (mean ± SD)	4 ± 2	5 ± 2	0.093
5′ Apgar score (mean ± SD)	6 ± 2	7 ± 2	0.272
RDS grade (mean ± SD)	3 ± 1	3 ± 1	0.064
Surfactant usage, *n* (%)	23 (50)	33 (38)	0.180
Intubation after birth, *n* (%)	20 (43)	24 (28)	0.064
Medicine for apnea, *n* (%)	36 (78)	80 (92)	0.025 *
Theophylline, *n* (%)	20 (43)	67 (77)	0.001 *
Caffeine, *n* (%)	16 (35)	13 (15)	0.001 *
Packed RBC transfusion, *n* (%)	36 (38)	59 (62)	0.205
Moderate and severe BPD, *n* (%)	20 (43)	31 (36)	0.376
PDA, *n* (%)	30 (65)	52 (60)	0.539
Without Tx, *n* (%)	6 (11)	21 (24)	0.130
With conservative Tx, *n* (%)	24 (52)	31 (36)	0.065
Late-onset sepsis, *n* (%)	16 (35)	25 (29)	0.473
Blood culture proved, *n* (%)	9 (20)	9 (10)	0.139
Clinical diagnosis, *n* (%)	7 (15)	16 (18)	0.645
Hypotension, *n* (%)	12 (26)	8 (10)	0.018 *
IVH ≥ grade III, *n* (%)	3 (7)	3 (3)	0.417
ROP ≥ stage III, *n* (%)	7 (15)	19 (22)	0.359
NEC ≥ stage II, *n* (%)	1 (2)	1 (1)	0.644

*, *p* value < 0.05; NCPAP, nasal continuous positive airway pressure; HFNC, high-flow nasal cannula; GA, gestational age; BBW, birth body weight; VD, vaginal delivery; NTISS, Neonatal Therapeutic Intervention Scoring System; pRBC, packed red blood cell; BPD, bronchopulmonary dysplasia; Tx, treatment; PDA, patent ductus arteriosus; IVH, intraventricular hemorrhage; ROP, retinopathy of prematurity; NEC, necrotizing enterocolitis.

**Table 2:** Respiratory outcomes and prognosis of infants undergoing NCPAP weaning with HFNC and room air groups.

Item	Mean ± SD		*p* Value
	Weaned by HFNC (*n* = 46)	Weaned by Room Air (*n* = 87)	
Length of Hospital Stay at Initiating NCPAP Weaning (days)	48 ± 28	34 ± 26	0.004 *
PMA at Initiating NCPAP Weaning (weeks)	35.6 ± 2.3	33.2 ± 2.5	<0.001 *
Duration of Weaning from NCPAP to HFNC in HFNC group or to Room Air in Room Air Group (days) ^#^	7 ± 10	20 ± 11	<0.001 *
PMA at Discontinuation of NCPAP (weeks)	36.6 ± 3.0	36.1 ± 2.4	0.291
Duration from Initiating NCPAP Weaning to Achieving Complete Room Air (days)	30 ± 21	20 ± 11	0.003 *
Length of Hospital Stay (days)	96 ± 38	78 ± 28	0.006 *
PMA at Discharge (weeks)	42.4 ± 4.1	39.4 ± 2.6	<0.001 *
Total Number of Days of Respiratory Therapy ^^^	80 ± 37	56 ± 25	<0.001 *

* *p* value < 0.05; ^^^ the total days of respiratory therapy support (included ventilator, NIPPV, NCPAP, HFNC) during admission; ^#^ the duration between starting weaning NCPAP and the discontinuation of NCPAP (days); NCPAP, nasal continuous positive airway pressure; HFNC, high-flow nasal cannula; PMA, post-menstrual age.

**Table 3:** Univariate regression analysis of associated factors which influenced PMA at initiating NCPAP weaning and the duration of weaning from NCPAP to HFNC in HFNC group or to room air in room air group (days).

Item	PMA at Initiating NCPAP Weaning (Weeks)				The Duration of Weaning from NCPAP to HFNC in HFNC Group or to Room Air in Room Air Group (Days)			
	Regression Coefficient	Lower 95% CI	Upper 95% CI	*p* Value	Regression Coefficient	Lower 95% CI	Upper 95% CI	*p* Value
Room air–HFNC ^@^	−2.324	−3.195	−1.453	<0.001 *	12.796	8.888	16.706	<0.001 *
GA	−0.129	−0.342	0.082	0.229	−0.211	−1.210	0.788	0.676
BBW	−0.001	−0.003	9.948	0.066	−0.001	−0.009	0.006	0.787
Female–Male ^!^	−0.063	−0.976	0.849	0.891	1.509	−2.759	5.777	0.486
C/S-VD ^#^	−0.348	−1.273	0.576	0.458	0.899	−3.436	5.235	0.682
NTISS	0.006	−0.056	0.068	0.856	0.183	−0.105	0.473	0.210
1′ Apgar score	−0.103	−0.349	0.144	0.412	0.550	−0.602	1.702	0.347
5′ Apgar score	−0.078	−0.307	0.151	0.503	0.210	−0.864	1.285	0.699
RDS grade	0.572	−0.073	1.218	0.082	−0.596	−3.652	2.461	0.701
Surfactant usage	1.367	0.474	2.261	0.003 *	−0.136	−4.463	4.191	0.950
Intubation after birth	0.666	−0.297	1.629	0.174	−1.365	−5.899	3.169	0.553
Apnea medicine								
Used–not used ^$^	−0.181	−1.584	1.221	0.799	5.611	−0.884	12.11	0.089
Caffeine- Theophylline ^^^	1.582	0.494	2.669	0.005 *	−6.759	−12.08	−1.442	0.013 *
pRBC transfusion	1.247	0.260	2.234	0.014 *	−1.858	−6.576	2.860	0.437
BPD ≥ moderate	−0.119	−1.058	0.818	0.801	−1.916	−6.297	2.466	0.389
PDA								
Without Tx	0.009	−1.125	1.144	0.987	−2.871	−8.159	2.417	0.285
Conservative Tx	1.543	0.656	2.430	<0.001 *	−4.588	−8.853	−0.323	0.035 *
Late-onset sepsis								
B/C proved	0.069	−1.265	1.403	0.919	2.644	−3.585	8.873	0.403
Clinical diagnosis	0.749	−0.449	1.949	0.218	−2.104	−7.742	3.533	0.462
Hypotension	−0.314	−1.564	0.936	0.620	−2.158	−8.005	3.689	0.467
IVH ≥ grade III	0.865	−1.328	3.059	0.436	−4.995	−15.25	5.262	0.337
ROP ≥ stage III	1.957	−0.858	3.057	0.001 *	0.032	−5.356	5.419	0.991
NEC ≥ stage II	0.314	−3.435	4.063	0.869	19.86	2.647	37.08	0.024 *

* *p* value < 0.05; ^@^ Compared room air group to HFNC group; ^!^ compared female to male; ^#^ compared C/S to VD; ^$^ compared used to not used; ^^^ compared Caffeine to Theophylline; CI, confidence interval; VD, vaginal delivery; C/S, cesarean section; NCPAP, nasal continuous positive airway pressure; HFNC, high-flow nasal cannula; GA, gestational age; BBW, birth body weight; VD, vaginal delivery; Tx, treatment. NTISS, Neonatal Therapeutic Intervention Scoring System; RDS, respiratory distress syndrome; B/C, blood culture; pRBC, packed red blood cell; BPD, bronchopulmonary dysplasia; PDA, patent ductus arteriosus; IVH, intraventricular hemorrhage; ROP, retinopathy of prematurity; NEC, necrotizing enterocolitis.

**Table 4:** Multiple regression analysis of associated factors which influenced PMA at initiating NCPAP weaning and the duration of weaning from NCPAP to HFNC in HFNC group or to room air in room air group (days).

	Item	Regression Coefficient	Lower 95% CI	Upper 95% CI	*p* Value	VIF
PMA at Initiating NCPAP Weaning (weeks)	Room air-HFNC ^@^	−1.592	−2.599	−0.586	0.002 *	1.158
	Surfactant usage	0.717	−0.227	1.661	0.135	1.177
	Caffeine-Theophylline ^^^	0.918	−0.216	2.053	0.112	1.289
	pRBC transfusion	0.636	−0.649	1.922	0.329	1.729
	PDA with Tx ^$^	0.961	0.003	1.919	0.049 *	1.202
	ROP ≥ stage III	0.119	−1.178	1.416	0.856	1.428
Duration of Weaning from NCPAP to HFNC in HFNC Group or to Room Air in Room Air Group (days)	Room air-HFNC ^@^	12.215	8.156	16.274	<0.001 *	1.134
	Caffeine-Theophylline ^^^	−0.974	−6.213	4.265	0.713	1.424
	PDA with Tx ^$^	−2.409	−6.577	1.758	0.255	1.282
	NEC ≥ stage II	21.569	6.542	36.598	0.005 *	1.018

* *p* value < 0.05; ^@^ Compared room air group to HFNC group; ^^^ compared Caffeine to Theophylline; ^$^ PDA with conservative treatment; NCPAP, nasal continuous positive airway pressure; HFNC, high-flow nasal cannula; CI, confidence interval, VIF, Variance Inflation Factor; Tx, treatment; PMA, post-menstrual age; pRBC, packed red blood cell; PDA, patent ductus arteriosus; ROP, retinopathy of prematurity; NEC, necrotizing enterocolitis.

**Table 5:** Univariate regression analysis of associated factors which influenced the duration from initiating NCPAP weaning to achieving complete room air and total number of days of respiratory therapy.

Item	Duration from Initiating NCPAP Weaning to Achieving Complete Room Air (Days)				Total Number of Days of Respiratory Therapy			
	Regression Coefficient	Lower 95% CI	Upper 95% CI	*p* Value	Regression Coefficient	Lower 95% CI	Upper 95% CI	*p* Value
Room air-HFNC ^@^	−10.468	−16.077	−4.858	<0.001 *	−24.194	−34.948	−13.440	<0.001 *
GA	0.003	−1.309	1.315	0.996	−9.884	−11.801	−7.966	<0.001 *
BBW	0.005	−0.005	0.014	0.356	−0.067	−0.082	−0.052	<0.001 *
Female-Male ^!^	−2.273	−7.869	3.324	0.423	−1.205	−12.187	9.777	0.829
C/S-VD ^#^	−4.952	−10.580	0.675	0.084	1.023	−10.119	12.166	0.856
NTISS	0.048	−0.333	0.429	0.805	0.712	−0.025	1.448	0.058
1′ Apgar score	−1.410	−2.907	0.087	0.065	−5.738	−8.537	−2.939	<0.001 *
5′ Apgar score	−0.806	−2.210	0.598	0.258	−4.594	−7.239	−1.948	<0.001 *
RDS grade	3.677	−0.284	7.639	0.069	9.489	1.807	17.171	0.016 *
Surfactant usage	1.259	−4.414	6.933	0.661	12.461	1.556	23.366	0.025 *
Intubation after birth	7.329	1.507	13.151	0.014 *	11.921	0.440	23.402	0.042 *
Apnea medicine								
Used–not used ^$^	−3.793	−12.385	4.799	0.384	1.492	−15.377	18.360	0.861
Caffeine- Theophylline ^^^	−2.264	−8.559	4.029	0.478	14.977	1.774	28.180	0.027 *
pRBC transfusion	1.758	−4.440	7.956	0.576	6.226	−5.875	18.327	0.311
PDA								
Without Tx	−4.144	−11.076	2.789	0.239	−8.179	−21.749	5.392	0.235
Conservative Tx	−3.557	−9.217	2.102	0.216	3.968	−7.155	15.091	0.482
BPD ≥ moderate	0.188	−5.578	5.954	0.949	−0.604	−11.891	10.683	0.916
IVH ≥ grade III	−6.193	−19.658	7.272	0.365	3.316	−23.120	29.753	0.804
Late-onset sepsis								
B/C proved	4.019	−4.146	12.185	0.332	2.634	−13.403	18.671	0.746
Clinical diagnosis	−2.392	−9.794	5.009	0.524	3.259	−11.242	17.760	0.657
Hypotension	3.863	−3.796	11.522	0.320	3.449	−11.589	18.488	0.651
ROP ≥ stage III	−3.335	−10.380	3.711	0.351	26.772	13.729	39.814	<0.001 *
NEC ≥ stage II	18.363	−4.453	41.178	0.114	−28.935	−73.752	15.882	0.204

* *p* value < 0.05; ^@^ Compared room air group to HFNC group; ^!^ compared female to male; ^#^ compared C/S to VD; ^$^ compared used to not used; ^^^ compared Caffeine to Theophylline; NCPAP, nasal continuous positive airway pressure; HFNC, high-flow nasal cannula; CI, confidence interval; GA, gestational age; BBW, birth body weight; VD, vaginal delivery; C/S, cesarean section; Tx, treatment; NTISS, Neonatal Therapeutic Intervention Scoring System; B/C, blood culture; RDS, respiratory distress syndrome; pRBC, packed red blood cell; BPD, bronchopulmonary dysplasia; PDA, patent ductus arteriosus; IVH, intraventricular hemorrhage; ROP, retinopathy of prematurity; NEC, necrotizing enterocolitis.

**Table 6:** Multiple regression analysis of associated factors which influenced the duration from initiating NCPAP weaning to achieving complete room air and total number of days of respiratory therapy.

	Item	Regression Coefficient	Lower 95% CI	Upper 95% CI	*p* Value	VIF
Duration from Initiating NCPAP Weaning to Achieving Complete Room Air (days)	Room air–HFNC ^@^	−9.549	−15.168	−3.931	0.001 *	1.026
	Intubation after birth	5.778	0.099	11.458	0.046 *	1.026
Total Number of Days of Respiratory Therapy	Room air-HFNC ^@^	−28.667	−37.396	−19.938	<0.001 *	1.261
	GA	−7.241	−10.129	−4.352	<0.001 *	2.827
	BBW	−0.031	−0.053	−0.009	0.004 *	2.874
	1′ Apgar score	−2.176	−6.357	2.005	0.305	4.567
	5′ Apgar score	2.055	−1.599	5.709	0.268	4.293
	RDS grade	3.522	−3.211	10.254	0.302	1.789
	Surfactant usage	0.906	−9.598	11.409	0.865	2.110
	Intubation after birth	−4.601	−13.973	4.771	0.333	1.475
	Caffeine-Theophylline ^^^	3.053	−6.319	12.426	0.519	1.274
	ROP ≥ stage III	0.830	−8.570	10.231	0.861	1.086

* *p* value < 0.05; ^@^ Compared room air group to HFNC group; ^^^ compared Caffeine to Theophylline; NCPAP, nasal continuous positive airway pressure; HFNC, high-flow nasal cannula; CI, confidence interval, VIF, Variance Inflation Factor; GA, gestational age; BBW, birth body weight; RDS, respiratory distress syndrome; ROP, retinopathy of prematurity.

**Table 7:** Univariate regression analysis of associated factors which influenced the length of hospital stay and the PMA at discharge.

Item	Length of Hospital Stay (Days)				PMA at Discharge (Weeks)			
	Regression Coefficient	Lower 95% CI	Upper 95% CI	*p* Value	Regression Coefficient	Lower 95% CI	Upper 95% CI	*p* Value
Room air-HFNC ^@^	−18.033	−29.616	−6.451	0.003 *	−3.044	−4.234	−1.853	<0.001 *
GA	−6.911	−9.298	−4.524	<0.001 *	−0.595	−0.866	−0.324	<0.001 *
BBW	−0.039	−0.058	−0.021	<0.001 *	−0.005	−0.007	−0.003	<0.001 *
Female-Male ^!^	1.929	−9.484	13.343	0.739	−0.216	−1.454	1.022	0.731
C/S-VD ^#^	−6.329	−17.823	5.204	0.279	−0.006	−1.263	1.251	0.992
NTISS	1.409	0.672	2.146	<0.001 *	0.035	−0.049	0.119	0.418
1′ Apgar score	−4.976	−7.940	−2.011	0.001 *	−0.603	−0.921	−0.284	<0.001 *
5′ Apgar score	−3.811	−6.606	−1.016	0.008 *	−0.479	−0.779	−0.179	0.002 *
RDS grade	10.305	2.335	18.274	0.012 *	1.135	0.271	1.999	0.010 *
Surfactant usage	11.148	−0.246	22.541	0.055	1.097	−0.142	2.337	0.082
Intubation after birth	32.621	21.886	43.356	<0.001 *	1.251	−0.046	2.549	0.059
Apnea medicine								
Used-not used ^$^	−7.408	−24.898	10.083	0.404	−0.639	−2.539	1.259	0.506
Caffeine- Theophylline ^^^	−0.805	−14.741	13.132	0.909	1.625	0.128	3.123	0.034 *
pRBC transfusion	1.179	−11.448	13.806	0.854	0.809	−0.554	2.173	0.242
PDA					0.554	−0.715	1.824	0.389
Without Tx	−5.501	−19.652	8.651	0.443				
Conservative Tx	−6.174	−17.710	5.361	0.292	−0.759	−2.292	0.774	0.329
BPD ≥ moderate	6.599	−5.078	18.278	0.266	0.710	−0.541	1.961	0.264
IVH ≥ grade III	−12.484	−39.887	14.919	0.369				
Late-onset sepsis					0.967	−0.835	2.769	0.291
B/C proved	−8.454	−25.067	8.159	0.316	−0.337	−1.973	1.299	0.684
Clinical diagnosis	1.909	−13.173	16.991	0.803	0.229	−1.468	1.926	0.789
Hypotension	−14.411	−29.857	1.036	0.067	−0.154	−3.137	2.827	0.919
ROP ≥ stage III	19.173	5.174	33.172	0.008 *	2.706	1.217	4.195	<0.001 *
NEC ≥ stage II	−22.763	−69.476	23.949	0.337	−2.163	−7.236	2.909	0.400

* *p* value < 0.05; ^@^ Compared room air group to HFNC group; ^!^ compared female to male; ^#^ compared C/S to VD; ^$^ compared used to not used; ^^^ compared Caffeine to Theophylline; NCPAP, nasal continuous positive airway pressure; HFNC, high-flow nasal cannula; CI, confidence interval; VD, vaginal delivery; C/S, cesarean section; Tx, treatment; GA, gestational age; BBW, birth body weight; RDS, respiratory distress syndrome; NTISS, Neonatal Therapeutic Intervention Scoring System; pRBC, packed red blood cell; BPD, bronchopulmonary dysplasia; PDA, patent ductus arteriosus; IVH, intraventricular hemorrhage; ROP, retinopathy of prematurity; NEC, necrotizing enterocolitis.

**Table 8:** Multiple regression analysis of associated factors which influenced length of hospital stay and the PMA at discharge.

	Item	Regression Coefficient	Lower 95% CI	Upper 95% CI	*p* Value	VIF
Length of Hospital Stay (days)	Room air-HFNC ^@^	−19.761	−29.393	−10.129	<0.001 *	1.141
	GA	−4.424	−7.916	−0.932	0.013 *	3.027
	BBW	−0.013	−0.038	0.012	0.301	2.770
	NTISS	0.759	0.105	1.412	0.023 *	1.254
	1′ Apgar score	−0.137	−4.789	4.516	0.954	4.019
	5′ Apgar score	3.069	−1.154	7.294	0.153	3.828
	RDS grade	−1.139	−7.893	5.616	0.739	1.211
	Intubation after birth	28.435	17.476	39.394	<0.001 *	1.445
	ROP ≥ stage III	12.059	0.153	23.966	0.047 *	1.212
PMA at Discharge (weeks)	Room air-HFNC ^@^	−3.284	−4.567	−2.001	<0.001 *	1.249
	GA	0.005	−0.421	0.431	0.979	2.816
	BBW	−0.006	−0.009	−0.003	<0.001 *	2.773
	1′ Apgar score	−0.210	−0.800	0.379	0.481	4.166
	5′ Apgar score	0.018	−0.506	0.542	0.945	4.042
	RDS grade	0.575	−0.215	1.365	0.152	1.129
	Caffeine-Theophylline ^^^	−0.072	−1.451	1.306	0.917	1.262
	ROP ≥ stage III	−0.448	−1.826	0.929	0.520	1.068

* *p* value < 0.05; ^@^ Compared room air group to HFNC group; ^^^ compared Caffeine to Theophylline; NCPAP, nasal continuous positive airway pressure; HFNC, high-flow nasal cannula; CI, confidence interval, VIF, Variance Inflation Factor; RDS, respiratory distress syndrome; GA, gestational age; BBW, birth body weight; ROP, retinopathy of prematurity.

## Data Availability

All data generated or analyzed during this study are included in the published article.
